# Multifaceted mechanisms of unconventional T cells in ischemia-reperfusion injury

**DOI:** 10.3389/fimmu.2026.1931069

**Published:** 2026-08-11

**Authors:** Jiajun Li, Peizhe Lin, Menghan Yuan, Yulin Wang, Hongyuan Liu, Ruo Jia, Jinghan Fan, Li Liang, Pingping Han, Qiujun Guo, Yuzhou He

**Affiliations:** 1 The Second Affiliated Hospital of Zhejiang Chinese Medical University, Hangzhou, China; 2 Zhejiang Chinese Medical University, Hangzhou, China; 3 Guang’anmen Hospital, China Academy of Chinese Medical Sciences, Beijing, China; 4 First Teaching Hospital of Tianjin University of Traditional Chinese Medicine, Tianjin, China; 5 College of Traditional Chinese Medicine, Tianjin University of Traditional Chinese Medicine, Tianjin, China

**Keywords:** ischemia-reperfusion injury, MAIT cells, NKT cells, sterile inflammation, unconventional T cells, γδ T cells

## Abstract

Ischemia-reperfusion injury (IRI) constitutes a common pathological basis for organ dysfunction across various critical clinical conditions, including ischemic stroke, myocardial infarction, and organ transplantation. This review focuses on the functions of unconventional T cells in IRI in the brain, liver, kidneys, heart, and mucosal barrier organs, highlighting their diverse regulatory mechanisms in various tissue injury patterns. It also examines the dual functions of these cells in IRI: instead of solely acting as consistent pro-inflammatory effectors, they demonstrate a context-dependent immunomodulatory profile influenced by the organ microenvironment and disease stage, capable of promoting inflammatory damage and facilitating anti-inflammatory repair. Their behavior is shaped by a common injury response program that integrates local antigen cues, the cytokine milieu, metabolic checkpoints, and signals from the repair stage. Expounding on the spatiotemporal heterogeneity, functional plasticity, and regulatory networks of unconventional T cells in IRI will enhance our understanding of the immunopathological mechanisms. It also offers a theoretical basis and potential targets for formulating precision immunomodulatory strategies tailored to specific time windows and cell subsets.

## 1 Introduction

Ischemia-reperfusion injury (IRI) refers to the secondary tissue damage that occurs following the restoration of blood flow and oxygen supply after a period of ischemia and hypoxia. This pathological process is observed in various clinical contexts, including myocardial infarction, ischemic stroke, organ transplantation, and acute kidney injury (1, 2). The primary pathological features include the abrupt onset of sterile inflammation and the disruption of immunological network homeostasis following reperfusion (2). In the ischemic phase, tissue hypoxia induces metabolic disturbances in cells; upon restoration of perfusion, the release of reactive oxygen species (ROS) and the secretion of damage-associated molecular patterns (DAMPs) incite immune cell infiltration and a cytokine storm, subsequently exacerbating endothelial barrier damage and extracellular matrix remodeling (3, 4). These events directly induce malfunction of the target organ and early graft failure in transplants, but in severe cases, they can precipitate systemic inflammatory response syndrome and multiple organ dysfunction syndrome (5, 6). IRI remains a major impediment limiting the efficacy of vascular recanalization therapies and the success of organ transplantation. Despite the recent continuous refinement of organ preservation techniques and reperfusion strategies, the development of effective therapies targeting the core mechanisms of IRI remains a critical therapeutic challenge.

Conventionally, early inflammation in IRI is predominantly caused by the rapid infiltration and activation of innate immune cells, with T lymphocytes primarily functioning as effectors of the adaptive immune response, sustaining inflammation in later-stage inflammation and contributing to tissue repair (7, 8). This theory fails to elucidate why the post-reperfusion inflammatory cascade can be initiated rapidly and thereafter amplified chronically. Reperfusion-related oxidative stress can rapidly establish a self-reinforcing inflammatory network (9). By contrast, the recruitment of conventional αβ T cells typically involves a delay, indicating that these cells are more likely to be essential contributors in the perpetuation of inflammation rather than its initial trigger (10). Therefore, research has increasingly focused on rapid effector lymphocytes with innate-like characteristics to elucidate the critical steps in the early recognition and enhancement of sterile inflammation (11, 12).

Unconventional T cells are distinguished from conventional αβ T cells by their antigen-recognition strategy, TCR structure, and response kinetics. Instead of recognizing peptide antigens presented by polymorphic major histocompatibility complex (MHC) molecules, they frequently respond to non-peptide or stress-associated ligands presented by non-classical antigen-presenting molecules, or sensed through MHC-independent mechanisms (13). The predominant cell types include γδ T cells, mucosal-associated invariant T (MAIT) cells, and natural killer T (NKT) cells (14). γδ T cells express γδ rather than αβ TCRs and recognize phosphoantigens, butyrophilin-dependent signals, non-classical MHC molecules, and tissue-derived danger signals, often through subset- and tissue-specific repertoires (15). MAIT cells bear a semi-invariant TCR and mainly detect microbial riboflavin-derived metabolites presented by MR1, with cytokine-driven activation also contributing during sterile inflammation (16). NKT cells co-express T-cell receptors and NK-lineage markers and are classically CD1d-restricted lipid antigen-reactive cells (17). Within this population, type I NKT cells, also termed invariant natural killer T (iNKT) cells, express a semi-invariant TCR and recognize glycolipid antigens. Whereas type II NKT cells, also referred to as diverse NKT (dNKT) cells, possess more diverse TCRs and respond to a broader range of self-lipid antigens (18).

Recent studies have demonstrated that these unconventional T cells can be activated extremely early in IRI and participate in the initiation and modulation of the inflammatory response (19, 20). Despite their shared innate-like properties, distinct subsets of unconventional T cells exhibit marked functional heterogeneity; during IRI, some subsets may exacerbate inflammatory injury, whereas others contribute to anti-inflammatory regulation and tissue repair (17). Although accumulating evidence has begun to reveal the important roles of unconventional T cells in IRI, a comprehensive understanding of their functional characteristics and regulatory networks remains incomplete. This complexity is largely influenced by the organ microenvironment, disease stage, and subset-specific properties. In this review, we summarize the activation pathways and immunomodulatory mechanisms of unconventional T cells in IRI, aiming to refine the immunopathological framework of IRI and identify potential targets for precision immunotherapeutic strategies based on these cell subsets.

## 2 Overview of IRI mechanisms

**Figure 1:**
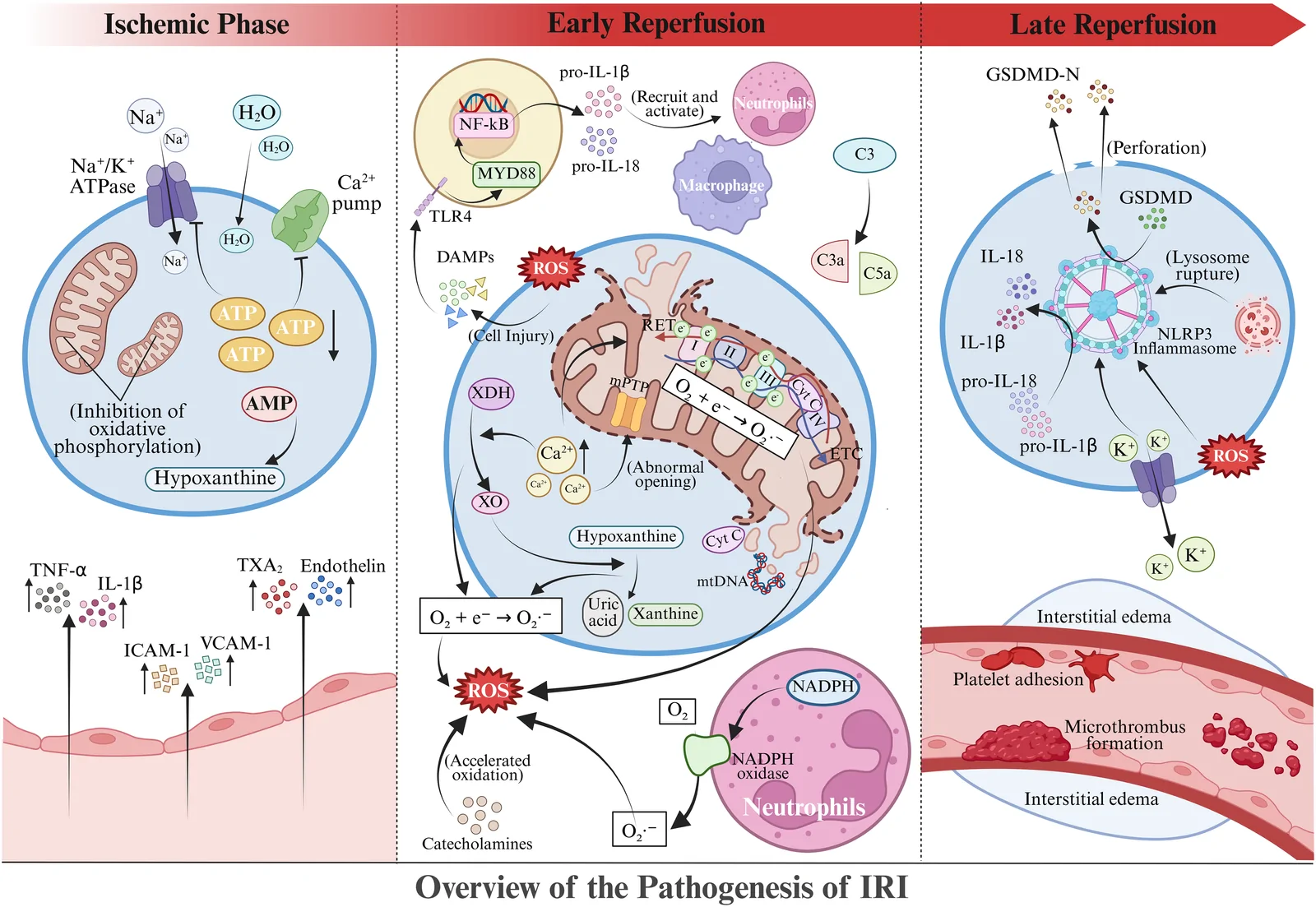
Overview of IRI pathogenesis. During the ischemic phase, restricted local blood flow results in tissue hypoxia, which suppresses mitochondrial oxidative phosphorylation and rapidly depletes intracellular ATP. Reduced energy availability impairs the activity of Na^+^/K^+^-ATPase and causes a decline in calcium pumps, causing cellular edema, disrupting ionic homeostasis, and making the cell increasingly prone to calcium overload. Concurrently, hypoxanthine accumulates, and ICAM-1, VCAM-1, TNF-α, IL-1β, and TXA_2_ expression are upregulated. Conversely, protective signals associated with nitric oxide, physiological anticoagulation, and vasodilation are diminished. In early reperfusion, mitochondrial complex damage, succinate accumulation, and RET promote a burst of ROS. Oxidative stress is further amplified by the conversion of XDH to XO, activation of neutrophil NADPH oxidase, and catecholamine oxidation. ROS mediate membrane lipid peroxidation, cytosolic and mitochondrial calcium overload, mitochondrial permeability transition pore (mPTP) opening, and the release of cytochrome c and mitochondrial DNA, and induce the release of DAMPs, including HMGB1 and ATP. Subsequently, DAMPs are recognized by TLR4, RAGE, and NLRP3, activating the MyD88, NF-κB, and inflammasome pathways, which facilitate leukocyte recruitment, IL-18 maturation, and GSDMD-dependent pyroptosis. The sustained generation of ROS and K^+^ efflux during reperfusion promotes activation of the NLRP3 inflammasome, accompanied by lysosomal rupture. Subsequently, pro-IL-1β and pro-IL-18 are cleaved into their mature forms, IL-1β and IL-18, which are subsequently released. In parallel, gasdermin D (GSDMD) is cleaved to generate the N-terminal fragment (GSDMD-N), which oligomerizes to form pores in the plasma membrane, thereby triggering pyroptotic cell death.

IRI affects the brain, heart, liver, kidneys, and other organs, but the temporal progression and pathological manifestations of ischemia and reperfusion differ considerably among tissues (21–23). To clarify the core pathogenesis of IRI, the following discussion is organized into three sequential phases: the ischemic phase, the early reperfusion phase, and the late reperfusion phase (**Figure 1**).

The ischemic phase is characterized by profound energy depletion and disruption of ionic homeostasis. Interruption of oxygen delivery halts mitochondrial oxidative phosphorylation, rapidly depleting intracellular ATP and impairing the function of sodium–potassium and calcium pumps. The resulting ionic disequilibrium promotes intracellular sodium and water accumulation, causing cellular edema and predisposing cells to subsequent calcium overload (24). Concurrently, degradation of adenine nucleotides causes hypoxanthine accumulation, providing abundant substrate for excessive generation of ROS upon reperfusion (25). Within the endothelium, ischemia upregulates the expression of adhesion molecules (intercellular adhesion molecule-1 [ICAM-1] and vascular cell adhesion molecule-1 [VCAM-1]).It also increases pro-inflammatory cytokines (including tumor necrosis factor [TNF]-α and interleukin [IL]-1β), and vasoconstrictor mediators (endothelin, thromboxane A2 [TXA2]), and simultaneously suppresses protective factors such as nitric oxide synthase, thrombomodulin, and prostacyclin (26, 27).

During the early reperfusion phase, once ROS surges, they directly damage membrane structures through lipid peroxidation and, together with impaired membrane transport, exacerbate cytosolic and mitochondrial calcium overload. Excessive accumulation of free intracellular calcium activates degradative enzymes, including calpains, dismantling cellular architecture. Additionally, it induces aberrant opening of the mitochondrial permeability transition pore (mPTP), resulting in dissipation of the mitochondrial transmembrane potential. As ATP synthesis ceases, mitochondria swell and rupture, releasing cytochrome c and mitochondrial DNA, intensifying the energy crisis and organelle damage (28). More critically, ROS-induced cellular injury stimulates the release of DAMPs—high mobility group box 1(HMGB1), ATP, mitochondrial DNA, and others—that are subsequently recognized by pattern recognition receptors on neighboring cells (TLR4, RAGE) and intracellular sensors, including NLRP3 (29, 30). TLR4 engagement activates the MyD88-dependent signaling, promoting nuclear translocation of the transcription factor NF-κB and initiating the transcription of pro-IL-1β and pro-IL-18 (31). Subsequently, this rapidly recruits and activates neutrophils and monocyte–macrophages, thereby amplifying the sterile inflammation response (32, 33). Concurrently, reperfusion induces cleavage and activation of complement component C3, liberating the anaphylatoxins C3a and C5a (34). Additionally, C5a, by binding to C5aR on leukocytes, significantly enhances leukocyte chemotaxis and the production of inflammatory mediators, including monocyte chemoattractant protein-1 (MCP-1), tumor necrosis factor-alpha (TNF)-α, interleukin (IL)-1, and IL-6, and the membrane attack complex (C5b-9) directly mediates cytolytic reactions, intensifying tissue injury (35).

Unlike the acute phase, the late phase of IRI represents a sustained and dynamically evolving pathological process that may persist for several days. Persistent oxidative stress, potassium efflux, and lysosomal rupture can act as a secondary signal that promotes oligomerization of intracellular NLRP3, followed by the recruitment of the adaptor protein ASC and pro-caspase-1 to assemble the inflammasome (36). Activated caspase-1 subsequently promotes the maturation of pro-IL-1β and pro-IL-18 and specifically cleaves gasdermin D (GSDMD) (37). The liberated N-terminal domain of GSDMD perforates the plasma membrane, inducing pyroptosis and allowing cellular contents and inflammatory cytokines to leak out (38). Under permissive conditions, this pyroptosis-driven amplification of inflammation may culminate in a cytokine storm, exacerbating endothelial dysfunction, DNA damage, and secondary tissue injury. Endothelial disruption, together with platelet adhesion and microthrombus formation, promotes capillary leakage and interstitial edema, sustaining microvascular perfusion deficits and the no-reflow phenomenon. These events impair the ability to restrict effective reperfusion, driving structural remodeling and progressive organ dysfunction (39). When the injury can be contained, regenerative pathways stimulate angiogenesis and extracellular matrix remodeling to restore function. If inflammation persists or the damage proves overwhelming, excessive fibroblast activation and collagen deposition ensue, leading to fibrosis and organ failure (40).

## 3 Physiological characteristics and basic functions of unconventional T cells

Unlike conventional αβ T cells, which recognize peptide antigens presented by polymorphic major histocompatibility complex (MHC) class I and II molecules, unconventional T cells employ distinct antigen-recognition modes. These cells primarily recognize non-peptide ligands, including glycolipids, vitamin B2 metabolites, and phosphoantigens, presented by non-classical MHC class I-like molecules, including CD1d and MR1, which present antigens to NKT cells and MAIT cells, respectively (16, 17). By contrast, γδ T cells recognize phosphoantigens through butyrophilin-dependent mechanisms (41, 42). Owing to the invariant or semi-invariant nature of their T cell receptors (TCRs), unconventional T cells are considered a critical immunological bridge between innate and adaptive immunity (**Tables 1**, **2**). In addition to TCR-dependent activation, liver-resident unconventional T cells can be stimulated through TCR-independent pathways, further reflecting their innate-like immunity (43). These cells rapidly sense environmental perturbations through expression of integrins and cytokine receptors, acquire pre-programmed effector functions during thymic development, and preferentially reside in mucosal barrier sites, including the liver and intestine, where they play unique roles in anti-infection, antitumor surveillance, and maintenance of tissue homeostasis and repair (14, 44, 45). Collectively, these characteristics confer diverse effector functions on unconventional T cells, enabling them to complement and extend the roles of conventional innate and adaptive immune cells in protective immunity, barrier maintenance, and tissue repair.

**Table 1:** Comparison of characteristics between conventional T cells and unconventional T cell subsets.

Comparison dimension	Conventional T cells (αβ T cells: CD4^+^/CD8^+^)	Unconventional T cell		
		γδ T cells	iNKT cells	MAIT cells
Core Phenotypic Markers	TCRαβ, CD3, CD4, and CD8	TCRγδ; representative subsets include human Vγ9Vδ2 and Vδ1, and mouse Vγ1^+^ and Vγ4^+^	Invariant TCRα chain, NK1.1(mouse)	Invariant TCRα chain (Vα7.2),CD161
Core Molecules for Antigen Recognition	Peptide antigens presented by MHC class I/II molecules	TCR-mediated recognition, engagement of multiple stress ligands/costimulatory receptors (NKG2D), and cytokine-mediated bypass activation	CD1d (lipid antigen-presenting molecule)	MR1 (which presents vitamin B metabolites)
Types of Target Antigens	Peptide fragments derived from protein antigens (processed from pathogens, tumors, or self-antigens)	Phosphoantigens, HSP60	Endogenous lipids, ceramides	Riboflavin metabolites, folate metabolites
Proportion Among Total T Cells in Human Peripheral Blood	60%–80%	3%–10% (predominantly Vδ2 subset)	0.001%–1%	1%–10%
Major Tissue Distribution	Peripheral blood, lymphoid tissues	Epithelium, dermis, spleen, liver, intestinal tract, peripheral blood	Lymphoid tissues, bone marrow, liver, lung, adipose tissue	Lymphoid tissues, lung, liver, gastrointestinal tract, female reproductive tract
Core Biological Functions	High antigen specificity; clonal expansion and immune memory; CD4^+^ T cell helper functions (via secretion of a broad spectrum of cytokines)/CD8^+^ T cell cytotoxic (killing) functions	Biphasic pro-inflammatory (IL-17A)/anti-inflammatory (TGF-β) functions	Biphasic pro-inflammatory (Interferon [IFN]-γ)/anti-inflammatory (IL-4) functions	Biphasic pro-inflammatory (IFN-γ)/mucosal protective functions

**Table 2:** Cytokines secreted by unconventional T cells and their functions.

Cytokines	Functional roles in IRI and tissue homeostasis	References
IL-17A	Acts as a major pathogenic driver in early IRI by disrupting tissue barriers, inducing cell apoptosis, and orchestrating massive neutrophil infiltration to enlarge infarct size.	(88, 147)
IFN-γ	Amplifies early tissue damage and microvascular dysfunction by inducing severe cytotoxicity and skewing macrophages toward a highly pro-inflammatory (M1) phenotype.	(164, 165)
IL-4 and IL-13	Exert tissue-protective effects during late reperfusion by driving the conversion of inflammatory macrophages into reparative (M2) phenotypes to resolve inflammation and promote repair.	(166, 167)
IL-10	Functions as a crucial immunoregulatory brake that suppresses excessive pro-inflammatory cytokine storms, dampens antigen presentation, and reduces destructive neutrophil recruitment.	(126, 168, 169)
TGF-β	Restrains excessive acute inflammation to enforce immune tolerance while critically regulating extracellular matrix deposition, fibroblast activation, and structural tissue remodeling.	(170, 171)
AREG	Acts as a potent epidermal growth factor-like mediator that directly stimulates epithelial cell proliferation to rapidly restore compromised mucosal and physical barrier integrity.	(172, 173)
KGF (FGF7) and FGF9	Essential paracrine growth factors that prevent apoptosis, drive rapid wound re-epithelialization (KGF), and initiate vital Wnt signaling loops for appendage regeneration (FGF9).	(174, 175)
IL-22	Maintains mucosal barrier homeostasis by promoting epithelial cell survival and regulating local antimicrobial peptide expression to prevent secondary infections in post-ischemic tissues.	(176, 177)

## 4 Organ-specific adaptations of unconventional T cells in IRI

### 4.1 Brain

Among the various subsets, γδ T cells are recognized as the major cellular source of IL-17A during the acute phase of cerebral ischemia (46, 47). Studies have demonstrated that in mouse models of ischemic brain injury, meningeal and brain-infiltrating γδ T cells are rapidly mobilized or functionally altered within the sterile inflammatory milieu (48). Under stimulation by IL-23 and IL-1β derived from dendritic cells and macrophages, these cells secrete large amounts of IL-17A through bystander activation mechanisms (49). This IL-17A synergizes with TNF-α released by microglia or macrophages to induce astrocyte expression of CXCL1 and other CXC/CC related chemokines, thereby promoting extensive neutrophil recruitment to ischemic regions and amplifying tissue damage (47, 50, 51). Furthermore, IL-17A acts on cerebral microvascular endothelial and glial cells to upregulate MMP-3, MMP-9, and adhesion molecule expression, further compromising blood-brain barrier integrity through neutrophil-mediated disruption of tight junctions (52, 53). Additionally, IL-17A promotes neutrophil extracellular trap (NET) formation through activation of the PKCζ–ERK–ROS–PAD4 signaling pathway, thereby exacerbating post-ischemic brain injury (54). Consistent with these findings, a multicenter randomized controlled study initially involving 136 mice demonstrated that anti-IL-17A treatment reduced MRI-measured infarct volume at day 3 after stroke. Of the 109 mice included in the final per-protocol primary endpoint analysis (57 anti-IL-17A, 52 IgG control), mean infarct volumes (± SD) decreased from 75.66 ± 34.79 mm³ to 61.77 ± 31.04 mm³ (55). Beyond neutrophil recruitment, γδ T cells modulate myeloid cell responses through IL-17A-mediated monocyte pathways. Ly6C^+^ monocytes express high levels of IL-17 receptors. IL-17A induces chemokines, including MCP-1/CCL2, RANTES/CCL5, and CXCL12, thereby promoting the migration of splenic and circulating monocytes into ischemic brain parenchyma and their polarization toward M1 macrophages—a process involving mTORC1-S6K1 axis activation (56). This early excessive inflammatory response releases neurotoxic substances, disrupts the blood-brain barrier, and exacerbates cerebral edema and secondary neuronal death (57). Conversely, during the later phase of injury, TGF-β secreted by M2 microglia suppresses mTOR signaling and promotes PPARγ-dependent M2 macrophage polarization, facilitating phagocytic clearance of tissue debris, secretion of neurotrophic and anti-inflammatory mediators, angiogenesis, and functional recovery in damaged brain tissue (58). Furthermore, γδ T cells exert direct cytotoxic capabilities, mediating apoptosis of neurons and brain-resident immune cells through perforin, granzymes, and death receptor pathways, including Fas/FasL or TRAILR (59). Inhibition of perforin-mediated neurotoxicity diminishes γδ T cell infiltration and enhances post-stroke functional recovery (59). IL-17A production after ischemic stroke may be influenced by the gut–brain axis; however, whether microbiota-dependent changes in meningeal IL-17A mainly reflect direct trafficking of intestinal γδT17 cells or indirect modulation of tissue-resident meningeal γδ17 cells remains incompletely defined. The study by Benakis et al. showed that antibiotic-induced alterations of the gut microbiota reduced intestinal IL-17^+^ γδ T cells, expanded regulatory T cells, and were associated with smaller infarct volumes in experimental stroke models (56). These findings have led to the hypothesis that gut-derived γδT17 cells, potentially guided by CCR6–CCL20 signaling, may contribute to post-stroke neuroinflammation through trafficking from intestinal lymphoid tissues to the meninges. Recent studies indicate that meningeal γδ17 cells are predominantly tissue-resident Vγ6^+^ RORγt^+^ populations, suggesting that microbiota-dependent regulation of IL-17A may also occur indirectly through modulation of resident meningeal γδ17 cell activation states, rather than exclusively through recruitment of intestinal γδT17 cells (48, 60). Moreover, the effects of antibiotics on γδT17 cells and stroke outcome may be influenced by segmented filamentous bacteria (SFB)-dependent immune conditioning (61), which represents an important confounding factor when interpreting antibiotic-based experiments.

Similar to γδ T cells, NKT cells respond rapidly in cerebral IRI; however, they can exacerbate inflammation or help restore immune balance. In experimental ischemic stroke models, iNKT cells translocate from the liver to the ischemic penumbra during the acute phase. Upon identifying endogenous lipid antigens provided by CD1d molecules or through IL-12 stimulation, they become activated and secrete IFN-γ and TNF-α, causing cytotoxic damage and exacerbating cerebral edema (62, 63). Moreover, the larger the infarct, the more pronounced the activation of iNKT cells in peripheral blood—indicating that their activation closely mirrors the severity of brain damage (64). In a permanent middle cerebral artery occlusion model, exogenous α-GalCer activation of iNKT cells was associated with an increase in the infarct volume from approximately 32% to 41% at 24 h (data presented as mean ± SD; n = 4–6 mice randomised per group, with 4–6 mice included in the final analysis) (65). These findings suggest that excessive iNKT cell activation may contribute to inflammatory cascade of cerebral IRI. However, iNKT cells assume a unique function in post-stroke immunosuppression. Clinical and animal studies have demonstrated that stroke-induced sympathetic excitation alters the functional program of peripheral iNKT cells, prompting them to secrete substantial amounts of IL-10 (66). Elevated IL-10 broadly suppresses antigen presentation by monocytes/macrophages and markedly impairs the chemotactic and bactericidal capabilities of effector cells, including neutrophils (67). This systemic immunosuppression may partially restrict immune responses against self-antigens, and it weakens antimicrobial defenses, increasing the risk of secondary infections like post-stroke pneumonia (68). Thus, improving stroke-associated iNKT dysfunction helps restore peripheral immune homeostasis and diminishes infection rates—further indicating that NKT cells participate in the focal inflammatory response of cerebral IRI but may influence long-term outcomes in patients with stroke by regulating systemic immune balance. Additionally, In a mouse cardiac-arrest/resuscitation model, dNKT cells trafficked to the brain, attenuated inflammatory responses and neuronal injury, and improved neurological outcomes, supporting a neuroprotective role for this NKT-cell subset in post-cardiac-arrest brain injury (69). These findings suggest that the effects of NKT cells may depend on the specific NKT-cell subset and the disease context.

**Figure 2:**
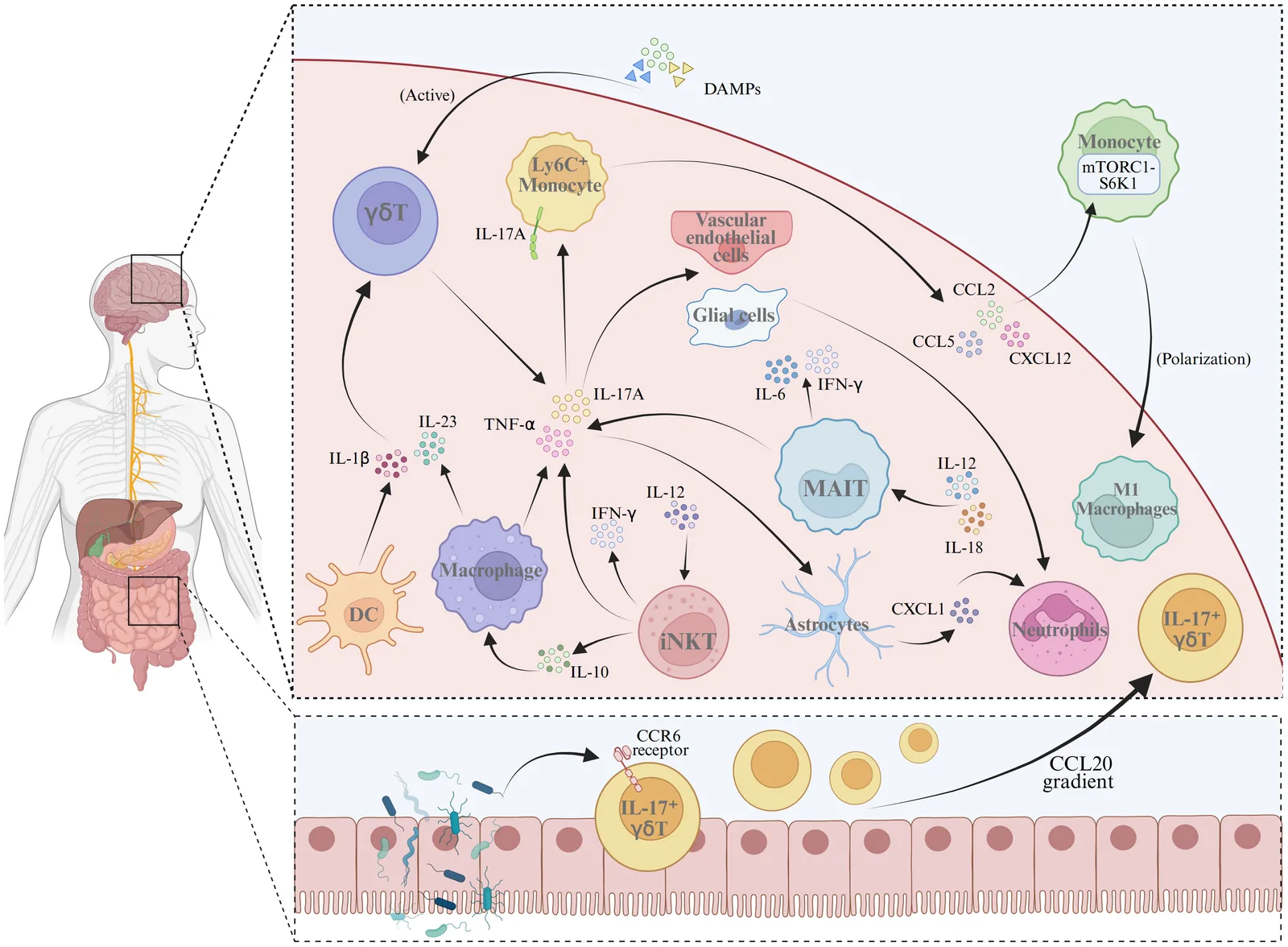
Mechanisms of inflammation amplification and immune imbalance mediated by unconventional T cells in cerebral IRI. DAMPs released from the ischemic tissue activate γδ T cells, NKT cells, and MAIT cells residing within the meninges and brain parenchyma. Stimulated by IL-23 and IL-1β derived from dendritic cells and macrophages, these γδ T cells produce large amounts of IL-17A. Acting in concert with microglia/macrophage-derived TNF-α, IL-17A induces astrocytes to express CXCL1 and other CXC/CC chemokines, facilitating neutrophil recruitment to the ischemic region. Additionally, IL-17A induces MCP-1/CCL2, RANTES/CCL5, and CXCL12 expression, facilitating the influx of Ly6C^+^ monocytes and polarizing them toward an M1 macrophage phenotype through the mTORC1–S6K1 axis, which aggravates cerebral edema and secondary neuronal death. The gut-brain axis may modulate this response; microbiota-dependent changes influence IL-17A-related neuroinflammation, although whether this mainly reflects direct trafficking of intestinal γδT17 cells, potentially guided by CCR6–CCL20 signaling, or indirect regulation of tissue-resident meningeal γδ17 cells remains unresolved. iNKT cells migrate to the ischemic penumbra in the acute phase, where they release IFN-γ and TNF-α upon CD1d-mediated lipid antigen presentation or IL-12 stimulation, intensifying cytotoxic injury and cerebral edema. Concurrently, stroke-associated sympathetic excitation drives iNKT cells to secrete IL-10, which inhibits peripheral antimicrobial immunity and increases the risk of secondary infection. MAIT cells are primarily activated and recruited to the ischemic brain tissue under the drive of IL-12 and IL-18; once there, they release IL-17A, IFN-γ, and IL-6, enhancing the inflammatory polarization of microglia/macrophages and the disruption of the blood–brain barrier.

In the sterile inflammatory context of cerebral ischemia, the activation of MAIT cells frequently depends on synergistic stimulation by cytokines such as IL-12 and IL-18 (70). Following ischemic onset, IL-12 and IL-18 are released, bind to receptors on the MAIT cell surface, and rapidly trigger their activation, inducing their migration from the periphery and meninges into the ischemic hemisphere. Upon arrival, they infiltrate the brain parenchyma and exacerbate ischemic brain injury by secreting IL-17, IFN-γ, and IL-6 (71). In patients with acute ischemic stroke, the frequency of circulating MAIT cells in peripheral blood declines shortly after onset and is closely associated with stroke severity and clinical prognosis by day 3 post-stroke (72). In a transient middle cerebral artery occlusion mouse model, genetic deletion of MR1 or pharmacological inhibition of MR1 with the inhibitory ligand i6-FP reduced infarct volume at 24 and 72 h, attenuated microglial activation, and lowered cerebral levels of IL-1β, IL-6, TNF-α, and IL-17A (73). These findings indicate that MR1-dependent MAIT-cell activation contributes to acute post-ischemic neuroinflammation in this experimental model. Upon activation, MAIT cells rapidly secrete IL-17A and TNF-α, which synergistically upregulate neutrophil chemokines, including CXCL1; facilitate inflammatory polarization of microglia and infiltrating macrophages; and compromise the blood–brain barrier, thereby exacerbating cerebral IRI (73, 74) (**Figure 2**).

### 4.2 Liver

NKT cells are a lymphocyte subset highly enriched in the liver. They possess NK cell receptors (NK1.1) and T cell receptors and play a significant role in the immunopathology of hepatic IRI. During the early reperfusion phase, ischemia-induced oxidatively modified lipids can function as self-antigens and be presented by CD1d, leading to the rapid activation of iNKT cells (75). Mice lacking CD1d or NK1.1 demonstrate significantly reduced hepatic necrosis and inflammatory infiltration, suggesting that CD1d-dependent iNKT activation is essential for the progression of IRI (76, 77). In specific warm hepatic ischemia models, intrahepatic iNKT cells rapidly proliferate post-reperfusion and generate substantial quantities of IFN-γ, with the dynamics of this growth paralleling the increase in serum ALT, thereby exacerbating liver tissue injury (76). IL-33–iNKT axis is particularly significant in this setting: in liver transplant recipients, serum IL-33 surges and peaks immediately after reperfusion, rising from 1.2 ± 1.1 pg/mL before transplantation to 77.5 ± 8.6 pg/mL. This surge is accompanied by the activation and hepatic recruitment of spleen-derived and intrahepatic iNKT cells, with the degree of activation positively correlating with early postoperative graft dysfunction (20). Additionally, IRF-1 helps maintain intrahepatic homeostasis of NK/NKT and CD8^+^ T cells by modulating the expression of IL-15/IL-15Rα complexes on hepatocytes and dendritic cells. IRF-1 deficiency results in a decrease in NKT cell populations and significantly mitigates liver injury in cold-ischemia IRI following allogeneic liver transplantation, highlighting the critical role of signaling axes that maintain NKT effector function in IRI pathogenesis (78). Conversely, type II NKT cells exhibit a more significant immunoprotective profile. Sulfatide can specifically activate type II NKT cells inducing a regulatory state before ischemia that suppresses IFN-γ secretion by iNKT cells and limits excessive myeloid cell recruitment, thus mitigating the increase in ALT and parenchymal necrosis following hepatic ischemia–reperfusion (79). Furthermore, the interaction between NKT cells and hepatic macrophages constitutes another critical layer of unconventional T cell-mediated IRI (80). In healthy liver tissue, activated iNKT cells can co-secrete IFN-γ with IL-4 and IL-13, directing macrophages from a pro-inflammatory toward a reparative phenotype and facilitating inflammation resolution and tissue regeneration (81, 82). In the context of steatotic liver disease, iNKT cells are more inclined to produce pro-inflammatory factors, including IFN-γ and IL-17, which promote the accumulation and activation of IL-1β^+^ and TNF-α^+^ macrophages, increasing the liver vulnerability to IRI (83). Specific deletion of CD1d in macrophages or enhancement of IL-13 signaling can partially reverse this inflammatory reprogramming, increasing the ratio of reparative macrophages and mitigating the severity of IRI in steatotic livers (80).

In the human liver, however, iNKT cells represent less than 1% of the T cell population, whereas MAIT cells are highly enriched, constituting 20%–40% of hepatic T cells (84). In patients with cirrhosis and end-stage liver disease, peripheral and intrahepatic MAIT cell populations are frequently significantly diminished; however, during acute IR stress, the residual MAIT cells exhibit a strongly activated phenotype (43, 85). Clinical evidence indicates that early portal vein reperfusion following liver transplantation induces upregulation of CD69 on MAIT cells, and the extent of activation positively correlates with peak serum IL-33 levels and liver function indicators (86). This indicates that, similar to iNKT cells, MAIT cells can be rapidly mobilized in an IL-33-dependent manner during ischemia-induced sterile inflammation, contributing to the early inflammatory response and hepatic functional decompensation.

**Figure 3:**
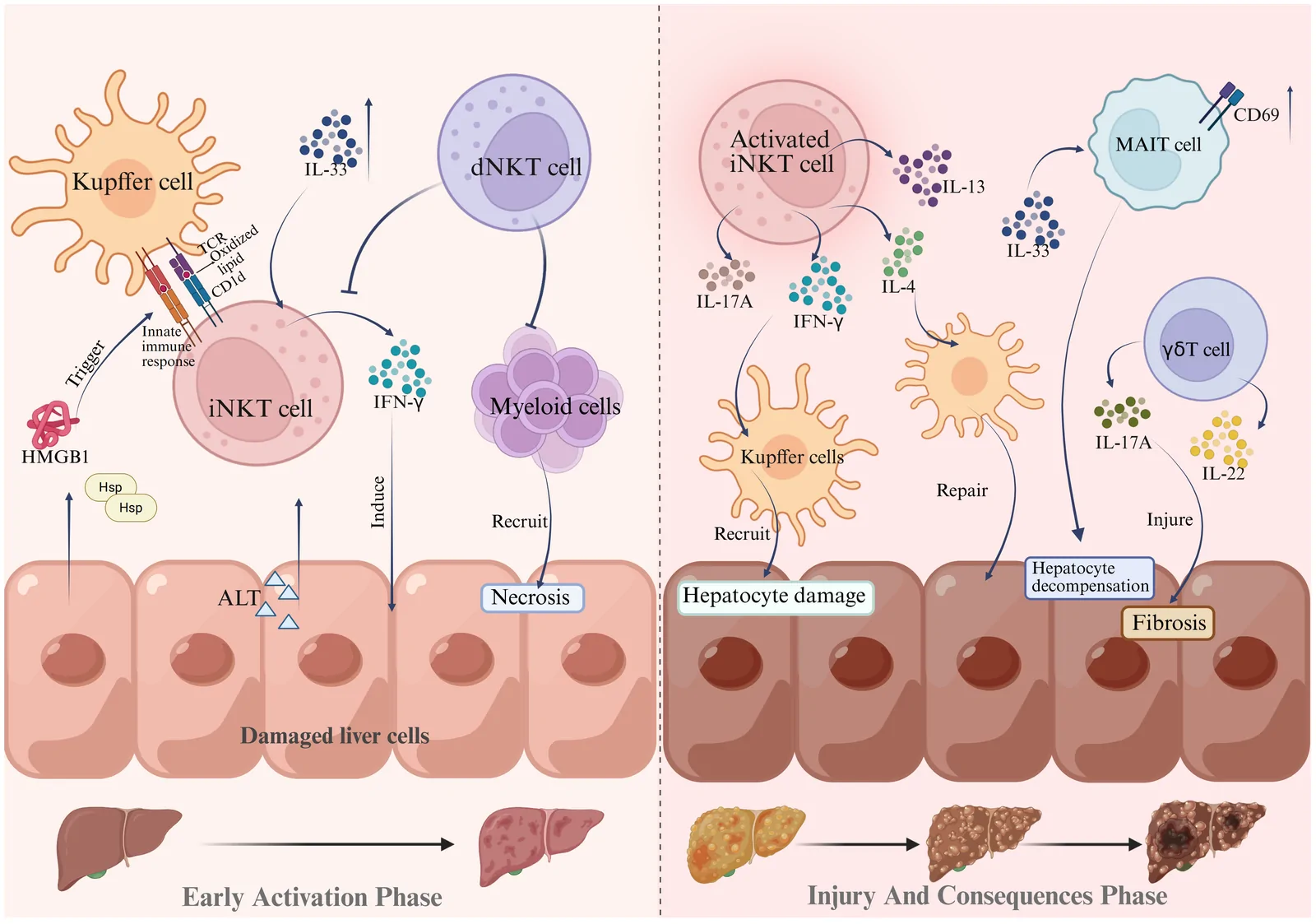
Pathogenic and protective immune mechanisms of unconventional T-cell subsets in hepatic IRI. This image illustrates the sterile inflammatory responses during hepatic IRI, initiated by DAMPs and amplified by unconventional T cell populations. During the ischemic phase, damaged hepatocytes release DAMPs, including HMGB1 and heat shock proteins (HSPs), and oxidatively modified lipids. Upon reperfusion, these signals, together with CD1d-mediated antigen presentation and increased IL-33, rapidly activate intrahepatic iNKT cells, MAIT cells, and γδ T cells. Among these populations, iNKT cells function as major early effectors, secreting large amounts of IFN-γ, thereby promoting the inflammatory cascade, hepatocyte necrosis, and inflammatory infiltration. MAIT cells exhibit CD69 upregulation following portal vein reperfusion, and the degree of their activation correlates with the peak of IL-33 and the severity of liver dysfunction, signifying their involvement in early inflammatory amplification. γδ T cells contribute predominantly through the secretion of IL-17A and IL-22, facilitating inflammatory cell recruitment, hepatocyte damage, and fibrosis progression—effects that are more pronounced in the setting of hepatic steatosis. Conversely, dNKT cells can be selectively activated by sulfatide and suppress IFN-γ secretion by iNKT cells and excessive myeloid cell recruitment, attenuating hepatic parenchymal necrosis.

Although γδ T cells are less abundant than NKT and MAIT cells in the liver, they can be rapidly activated during hepatic IRI and enhance the inflammatory response by secreting IL-17A and IL-22, which contribute to hepatocyte injury and fibrosis (87). In mouse models of hepatic IRI, γδ T cells participate in the initial inflammatory response and exacerbate liver damage; specifically, RORγt^+^/IL-17A-associated unconventional T cells are a major source of IL-17A during IRI, and the blockade of γδ T cell receptors or IL-17A alleviates hepatic inflammatory infiltration and injury (88). In a model of IRI associated with steatotic liver transplantation, increased infiltration of γδ T cells in the IRI group enhances inflammatory signaling through IL-17A secretion, exacerbating hepatocyte damage and fibrotic progression (89). The administration of anti-γδTCR or anti-IL-17A antibodies significantly attenuates liver injury and inflammatory cell infiltration, suggesting that γδ T cells contribute to hepatic IRI injury through IL-17A downstream signaling pathways (90) (**Figure 3**).

### 4.3 Kidney

In renal IRI, iNKT cells are triggered upon recognizing lipid antigens presented by CD1d molecules on dendritic cells, subsequently secreting substantial quantities of IFN-γ (91). The localized increase in IFN-γ stimulates tubular epithelial cells and vascular endothelial cells to secrete chemokines, including CXCL1 and MIP-2, which facilitate neutrophil infiltration. During the subsequent respiratory burst and degranulation, neutrophils release ROS and proteolytic enzymes that directly damage the tissue (92, 93). Simultaneously, IFN-γ, combined with TNF-α, IL-1β, and other pro-inflammatory mediators, acts on tubular epithelial cells, upregulating inducible nitric oxide synthase, resulting in the production of highly toxic peroxynitrite that disrupts mitochondrial function (94, 95). Additionally, it activates death receptor pathways such as Fas/FasL to initiate apoptosis and necrotic programs, thereby exacerbating tubular injury and functional decline (96). In renal IRI, A2AR signaling in CD11c^+^ dendritic cells limits tissue injury by suppressing dendritic-cell-mediated activation of CD1d-restricted iNKT cells and their IFN-γ response. Pharmacologic A2AR activation reduces the renal accumulation of IFN-γ-producing CD1d tetramer-positive NKT cells after ischemia–reperfusion. Moreover, adoptive transfer of A2AR agonist-conditioned, α-galactosylceramide-loaded dendritic cells attenuates tubular injury and renal dysfunction (97). Mechanistically, A2AR activation downregulates activating costimulatory molecules, including CD40 and OX40L, while upregulating the inhibitory ligand B7-DC, thereby generating a more tolerogenic dendritic-cell phenotype without substantially altering CD1d-mediated glycolipid antigen presentation. These findings support a model in which tubular epithelial stress and endothelial activation create a pro-inflammatory milieu, whereas CD1d-dependent lipid antigen presentation by dendritic cells, together with activating costimulatory signals, promotes a Th1-skewed iNKT-cell response characterized by IFN-γ production and neutrophil recruitment. By contrast, adenosine-A2AR signaling in CD11c^+^ dendritic cells acts as a local regulatory brake on this response. Thus, during early renal IRI, iNKT cells appear predominantly pathogenic when antigen presentation and activating costimulatory signals outweigh adenosinergic restraint. Separately, HIF-2α has been identified as a negative regulator of NKT-cell cytotoxicity during renal IRI, primarily based on evidence from mouse models (98). In contrast to the pathogenic role of iNKT cells, sulfatide-reactive type II NKT cells exert renoprotective effects by producing IL-10 and IL-4, thereby promoting the survival of hypoxic tubular epithelial cells. This protective effect has been demonstrated using human type II NKT cells *in vitro* together with mouse models of renal IRI (99). Reduced tubular epithelial injury may consequently limit DAMP release, thereby attenuating subsequent innate immune activation (100). However, activated type II NKT cells can induce iNKT cells to become anergic, resulting in reduced IFN-γ secretion. Renal biopsies from patients with acute tubular necrosis demonstrate an inverse correlation between the quantity of type II NKT cells and disease severity, highlighting their role in tissue protection and repair (101).

Notably, single-cell RNA sequencing of human kidney tissue identified an immune-cell cluster annotated as NK-T cells on the basis of the co-expression of T-cell genes, including CD3D and CD3E, and NK-associated genes, including KLRD1 and NKG7. In ischemic acute kidney injury, PALM3 and CD46 were among the genes upregulated in this cluster, indicating an altered inflammatory and complement-related transcriptional profile (102). Because this classification was based on transcriptomic clustering, these findings do not, by themselves, establish a causal role for PALM3 or CD46 in NKT-cell-mediated renal IRI. Given the reported associations of PALM3 with TLR4-related inflammatory signaling and the immunomodulatory functions of CD46 (103, 104), the PALM3- and CD46-associated inflammatory and complement pathways may represent candidate pathways for future investigation. Whether PALM3 directly regulates TLR4–MyD88/TRIF–NF-κB/IRF3 signaling in renal NKT cells, or whether CD46 expression functionally contributes to altered complement regulation in this setting, remains to be determined. More broadly activated macrophages, monocytes, dendritic cells, and B cells, together with these signaling pathways, form an inflammatory network that propels local immune-inflammatory cascades and advances tissue injury, representing important cellular sources of the inflammatory phenotype in ischemic kidney injury (105).

Furthermore, γδ T cells in renal IRI primarily exhibit pro-inflammatory and pro-injury features. In the acute phase of renal IRI, γδ T cell infiltration occurs in a highly synchronized manner both temporally and spatially with the tubular release of HMGB1 (106). Increased HMGB1 levels in renal tissue and peripheral blood serve as a potent danger signal from ischemic tissue to the immune system, enhancing inflammation through the TLR4 pathway and promoting the activation and proliferation of IL-17^+^ γδ T cells, thus exacerbating local inflammation (107). Simultaneous clinical observations reveal that increased levels of urinary cell cycle arrest biomarkers (including tissue inhibitor of metalloproteinases-2 and insulin-like growth factor binding protein 7) in patients with renal IRI correlate with a significant decrease in peripheral blood γδ T cell counts (106). This discovery, along with observations in mouse models indicating a concomitant increase in renal γδ T cell numbers and a decline in peripheral blood counts, implies that stress signals may induce directional migration of γδ T cells from the peripheral circulation to the damaged kidney. Contrary to conventional perspectives, recent evidence indicates that renal-infiltrating γδ T cells may not directly regulate neutrophil recruitment; rather, they facilitate the recruitment and activation of adaptive immune cells such as CD4^+^ T cells through the secretion of cytokines like IL-17A, thus sustaining local inflammation (108, 109). Mouse experiments have further corroborated this perspective. Deleting γδ T cells does not significantly alter serum creatinine levels during the acute phase of IRI (24 h); however, it attenuates tubular necrosis at subsequent stages, enhances renal function recovery, and lowers mortality (110). This suggests that γδ T cells predominantly regulate the continuation of inflammation rather than the initial phase of injury. Collectively, these findings confirm that γδ T cells assume a critical pathogenic role in sustaining inflammation and advancing the progression of tissue damage in renal IRI.

**Figure 4:**
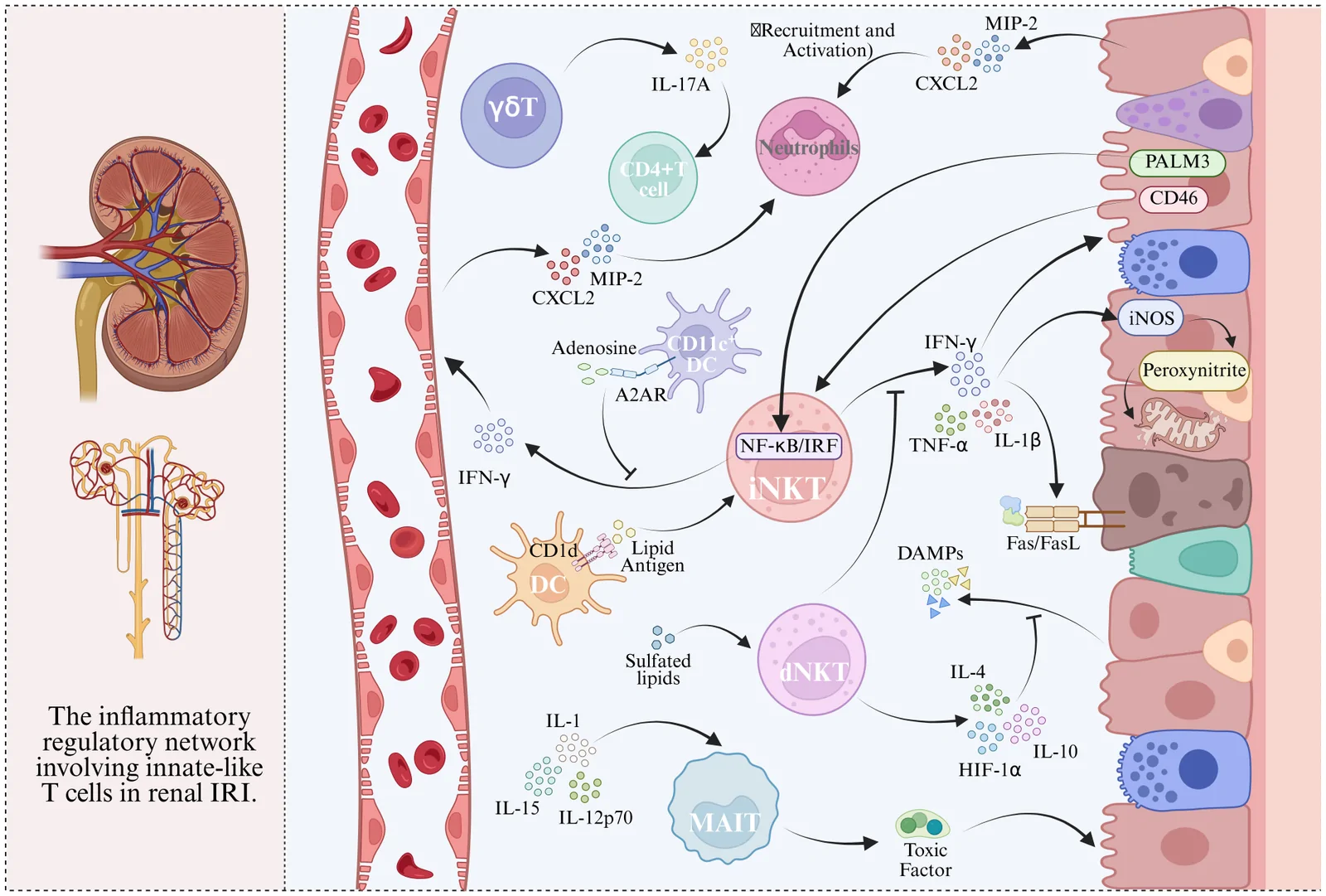
The inflammatory regulatory network involving unconventional T cells in renal IRI. Dendritic cells stimulate iNKT cells through CD1d-mediated presentation of lipid antigens, inducing substantial IFN-γ release. IFN-γ further stimulates renal tubular epithelial cells and endothelial cells to produce chemokines, promoting neutrophil infiltration and mediating tissue damage through respiratory burst, degranulation, and release of ROS and proteolytic enzymes. Simultaneously, IFN-γ synergizes with TNF-α and IL-1β to upregulate iNOS, promoting peroxynitrite formation and mitochondrial dysfunction, and activate the Fas/FasL death receptor pathway to induce apoptosis and necrosis. Adenosine-A2AR signaling in CD11c^+^ dendritic cells (DCs) limits CD1d-restricted iNKT-cell activation and IFN-γ production, thereby attenuating renal IRI. Conversely, dNKT cells activated by sulfatide upregulate HIF-1α, IL-10, and IL-4, facilitating renal tubular epithelial cell proliferation, reducing apoptosis and DAMPs release, and inducing iNKT cell anergy. Aberrant expression of the NKT cell-associated molecules PALM3 and CD46 may mediate TLR4-related inflammatory signaling and complement amplification, respectively. γδ T cells infiltrate in synchrony with HMGB1 release and, upon activation through the TLR4 axis, secrete IL-17A, enhancing recruitment of adaptive immune cells, including CD4^+^ T cells, and sustaining persistent inflammation. MAIT cells are activated under hypoxic conditions in the presence of IL-12p70, IL-15, and IL-1β, releasing cytotoxic molecules and inducing renal tubular epithelial cell necrosis.

The function of MAIT cells in renal IRI merits consideration. Although MAIT cells are comparatively rare in mice, they are more prevalent in the human kidney and are highly sensitive to hypoxia (70). In an *in vitro* co-culture model replicating the renal fibrotic/hypoxic microenvironment, hypoxic renal tubular epithelial cells markedly enhanced MAIT cell activation in the presence of inflammatory cytokines, including IL-12p70, IL-15, and IL-1β, inducing MAIT cells to release cytotoxic molecules and facilitate renal tubular epithelial cell necrosis (111). This indicates that MAIT cells may represent an essential driver of tubular injury under ischemic and hypoxic conditions (**Figure 4**).

### 4.4 Heart

In the pathological progression of cardiac IRI, γδ T cells, a lymphocyte population that bridges innate and adaptive immunity, are rapidly activated during the initial ischemic period. Despite constituting 1%–5% of peripheral T lymphocytes, γδ T cells are a significant source of local IL-17A in myocardial infarction and reperfusion injury (112). This pathogenic activation is not isolated; rather, it is closely regulated by the local microenvironment. Early-responding macrophages upregulate the expression of inflammasome component ASC to enhance IL-1β production, and in conjunction with IL-23 produced by M1 macrophages and neutrophils within the infarct, this stimulates γδ T cells to secrete IL-17A (113). The resulting surge in IL-17A enhances neutrophil recruitment, exacerbates tissue damage, and eventually induces cardiomyocyte apoptosis (114). Simultaneously, the body engages endogenous regulatory mechanisms to prevent excessive inflammatory escalation. Pentraxin 3 (PTX3) is an innate immune molecule from the long pentraxin family of soluble humoral pattern recognition receptors. It is rapidly induced in neutrophils, monocytes/macrophages, dendritic cells, and endothelial cells upon stimulation with TLR ligands, IL-1β, or TNF, and plays a role in regulating inflammation by modulating inflammatory cell recruitment and tissue repair (115, 116). In a mouse model of myocardial infarction, PTX3 deficiency exacerbates myocardial injury, whereas PTX3 confers a cardioprotective effect by inhibiting the IL-23/IL-17 axis derived from myeloid cells, thereby limiting the proliferation of γδ T cells (117). The tissue-damaging activity of γδ T cells is rigorously regulated by Tregs (118). Tregs directly inhibit γδ T cell activation through cytokines such as IL-10 and TGF-β, limiting their infiltration into injured tissue (119). In the absence of Treg-mediated immunosuppression, however, γδ T cells unleash IFN-γ, a crucial effector cytokine, thereby amplifying the inflammatory cascade (120). In a rat syngeneic heart transplantation I/R model, after 8 h of ischemia and subsequent reperfusion, the graft myocardium demonstrates extensive infiltration by γδ T cells with high NKG2D expression. Blockade using an anti-NKG2D monoclonal antibody significantly decreases serum troponin T levels, diminishes neutrophil infiltration, downregulates TNF-α and ICAM-1 mRNA expression in the graft tissue, and enhances cardiac functional indices, indicating effective attenuation of I/R injury (121). However, γδ T cells are not solely pathogenic. In the cardiac repair phase, they secrete IL-10, IL-4, and other cytokines that promote M2 macrophage polarization, thereby actively contributing to post-injury tissue repair (122). In a hindlimb ischemia model, complete depletion of γδ T cells mitigates acute inflammation; however, it also leads to an insufficient transition of macrophages from an M1-like to an M2-like phenotype, disrupts repair-associated pathways, including TIMP-1/pro MMP-9, and compromises angiogenesis and tissue regeneration (123).

In the hyperacute phase of I/R, NKT cells exhibit a pathogenic phenotype characterized by Th1-type inflammation (124). However, the injured myocardium rapidly establishes a repair-oriented niche characterized by regulatory cytokines, Treg interaction, macrophage reprogramming, and vascular remodeling signals (125). In specific activation modes, for instance, stimulation with potent glycolipid antigens such as α-GalCer and within particular tissue milieus, NKT cells can induce immunoregulatory factors such as IL-10, IL-4, and IL-13, thereby limiting excessive inflammation and supporting favorable ventricular remodeling (126, 127). In cardiac IRI, activated iNKT cells infiltrate the infarcted myocardium and facilitate favorable left ventricular remodeling (128). Clinical data indicate that patients with acute myocardial infarction have low absolute counts of circulating iNKT cells following emergency reperfusion therapy. Subsequent studies establish the circulating iNKT cell count as an independent predictor of long-term outcomes, including restenosis (129), suggesting a protective role for iNKT cells in mitigating cardiac IRI and facilitating subsequent vascular repair. Mechanistic experiments further illustrate that Tregs can inhibit excessive NKT cell activation and IFN-γ production through a contact-dependent mechanism, whereas NKT cells, in turn, secrete IL-2 to promote Treg survival, thereby establishing a reciprocal regulatory association in which the two populations simultaneously restrain and sustain one another (130, 131). Clinical studies demonstrate that subsets of sulfatide-specific type II NKT cells can migrate from the peripheral blood into the brain within 6–24 h post-resuscitation and accumulate in the hippocampus. These cells primarily express TGF-β1 and IL-10, with negligible levels of perforin, IFN-γ, and TNF-α, demonstrating an overall immunosuppressive and anti-inflammatory phenotype (69). These findings raise the possibility that a comparable NKT subset transition, from a pro-inflammatory toward a more immunoregulatory phenotype, may also occur during cardiac IRI. However, whether such a subset shift exists in the injured myocardium and contributes to cardiac repair remains to be experimentally validated.

**Figure 5:**
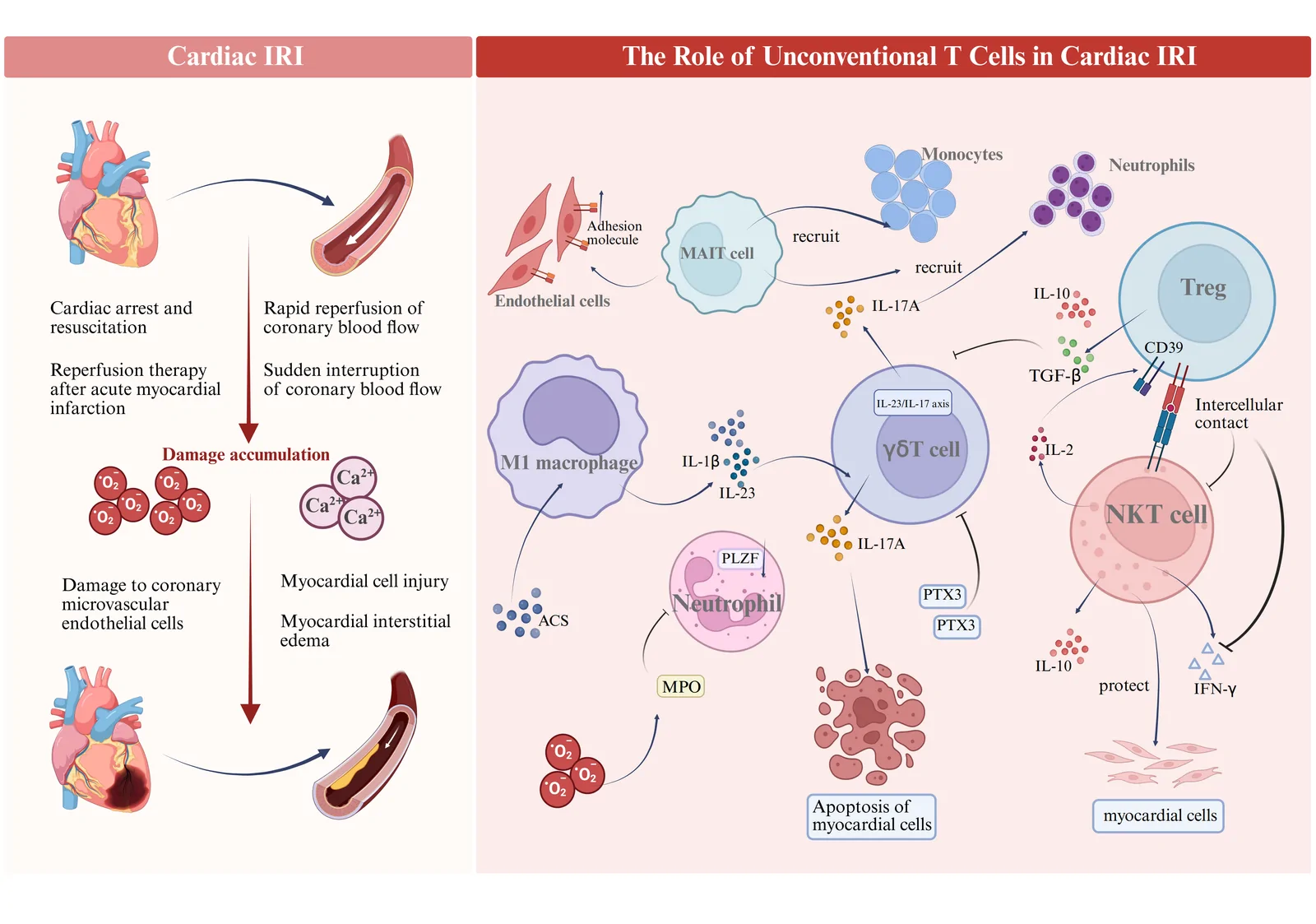
Schematic diagram of inflammatory amplification and tissue repair mechanisms involving unconventional T cells in cardiac IRI. Following abrupt cessation and subsequent restoration of coronary blood flow, cardiomyocytes are first subjected to oxidative stress, calcium overload, mitochondrial dysfunction, and disrupted energy metabolism, alongside endothelial damage and myocardial interstitial edema. Damaged tissues release inflammatory mediators, including TNF-α, IL-1β, and IL-6, driving the infiltration of neutrophils and monocytes/macrophages into the ischemic myocardium. During the early inflammatory phase, macrophages produce IL-1β through the ASC-associated inflammasome, which cooperates with IL-23 derived from M1 macrophages and neutrophils to activate γδ T cells to secrete IL-17A. IL-17A, in turn, facilitates further neutrophil recruitment, amplifying the local inflammatory cascade and aggravating cardiomyocyte apoptosis and tissue injury. In parallel, counter-regulatory immune mechanisms are engaged: PTX3 inhibits the myeloid cell-driven IL-23/IL-17 axis, limiting γδ T cell expansion, whereas Tregs restrain γδ T cell activation and their infiltration into injured areas through IL-10 and TGF-β. NKT cells largely adopt a pathogenic Th1-type phenotype during the hyperacute phase of IRI, yet under specific modes of activation and within the local microenvironment, they can produce immunoregulatory factors, including IL-10, IL-4, and IL-13, helping to control inflammation and enhance left ventricular remodeling. MAIT cells may exacerbate cardiac IRI outcomes by secreting IL-17, IFN-γ, and TNF-α, upregulating ICAM-1 and VCAM-1, and inducing CXCL8 and CCL2 release, fueling coronary microvascular inflammation and disrupting endothelial homeostasis.

Compared with γδ T and NKT cells, the evidence for a role of MAIT cells in cardiac I/R remains insufficient. Whole-blood RNA sequencing in patients with INOCA revealed an expression pattern enriched for transcripts associated with MAIT cells, plasmacytoid dendritic cells, and memory B cells, together with reduced neutrophil-associated transcripts. ZBTB16, which encodes the MAIT-associated transcription factor PLZF, was decreased despite increases in several other MAIT-related transcripts (132). These findings indicate that MAIT cells may indirectly exacerbate myocardial injury following ischemia–reperfusion by amplifying coronary microvascular inflammation and disrupting endothelial homeostasis. In myocardial I/R studies, activated MAIT cells rapidly produce IL-17, IFN-γ, and TNF-α, facilitating the recruitment of neutrophils and monocytes to the ischemic myocardium, upregulating surface expression of ICAM-1 and VCAM-1, and inducing the release of various chemokines such as CXCL8 and CCL2, thereby worsening coronary microvascular inflammation and endothelial dysfunction (133, 134). Furthermore, persistent effector molecules such as IL-17 and IFN-γ inhibit the growth of nascent capillaries and inappropriately activate cardiac fibroblasts. These findings suggest that MAIT cells affect microvascular homeostasis. Future mechanistic studies focusing on their functional regulation may facilitate the development of novel intervention strategies and yield significant advancement in associated immunotherapies (**Figure 5**).

### 4.5 Other organs

In contrast to sterile solid organs such as the heart, kidney, and brain, the intestine and lung, which engage directly with the external environment and microbial communities through their mucosal surfaces, have unique immunopathological mechanisms in IRI (135, 136). In IRI and associated disease processes in the intestine and lung, ischemia-induced barrier dysfunction is frequently accompanied by translocation of intestinal microbiota-derived products. Unconventional T cells, including γδ T cells and NKT cells, function as early initiators of local inflammatory responses and key mediators of immune signaling cascades in distant organs through complex inter-organ communication networks such as the gut-brain axis and the gut-lung axis (137).

In intestinal IRI, the pathological process is characterized by rapid neutrophil recruitment, upregulated expression of pro-inflammatory cytokines, and the potential for secondary injury to distant organs, including the liver. γδ T cells, which constitute a major subset of intestinal intraepithelial lymphocytes, exert a prominent pro-inflammatory effect in this context. Previous studies have demonstrated that, in an acute intestinal IRI model, activation of γδ T cells promotes IL-17A production, thereby facilitating neutrophil recruitment and infiltration (138). Furthermore, specific deletion of γδ T cells significantly reduces local pro-inflammatory cytokine expression, neutrophil infiltration, and serum transaminase levels (137), highlighting their critical role in mediating the acute inflammatory response during intestinal IRI. In contrast to the pro-inflammatory functions of γδ T cells, NKT cells play a protective role: activated NKT cells upregulate anti-inflammatory cytokines, including IL-4 and IL-10, thereby improving intestinal barrier integrity and mitigating intestinal injury (139, 140). Meanwhile, MAIT cells regulate the local immune response by secreting factors such as IL-10, reinforcing the intestinal barrier and consequently mitigating damage to distant organs (71, 141).

Unconventional T cells likewise function as critical immune regulators in pulmonary IRI, a condition commonly encountered in lung transplantation, and in brain-induced lung injury. Lung IRI is typically characterized by pulmonary edema, neutrophil infiltration, and the release of pro-inflammatory mediators (142). Within this pathological context, γδ T cells have been identified as a major source of IL-17 in the lung, and the extent of their infiltration correlates positively with the severity of transplant-related injury (143). Through IL-17A secretion, γδ T cells promote neutrophil recruitment, thereby contributing to alveolar edema and impaired pulmonary function. Conversely, therapeutic hypercapnia mitigates post-transplant lung injury by dampening γδ T cell activation and IL-17 release (144). Additionally, NKT cells contribute to the pro-inflammatory milieu in lung IRI, with studies demonstrating that iNKT cells tend to exacerbate ischemic damage by secreting IL-17A and recruiting large granular lymphocytes, a pattern similar to their pathogenic mechanisms in renal and hepatic IRI (75, 145, 146).

### 4.6 Shared pathways and organ-specific determinants of unconventional T-cell responses in IRI

Ischemia and reperfusion generate shared upstream signals, including DAMP release, oxidative stress, and endothelial activation. The responses of iNKT, γδ T, and MAIT cells are subsequently shaped by tissue-dependent antigen presentation, the local cytokine and alarmin milieu, metabolic checkpoints, and dominant myeloid-cell interactions.

Across organs, several recurrent but context-dependent patterns emerge. γδ T-cell-derived IL-17A represents a recurring inflammatory module in the brain, liver, heart, intestine, and lung, whereas renal γδ T cells appear to contribute more prominently to the persistence of inflammation and adaptive immune-cell recruitment than to the initiation of neutrophil influx. NKT-cell polarity is determined by the balance between activating and restraining signals: oxidized lipids and IL-33 favor inflammatory iNKT-cell responses in the liver; in the kidney, CD1d-dependent activation by dendritic cells is counterbalanced by A2AR signaling in CD11c^+^ dendritic cells, which limits activating costimulation and IFN-γ production; whereas Treg interactions, macrophage reprogramming, and repair-associated cytokines can shift cardiac NKT-cell responses toward immunoregulation and remodeling. Evidence for MAIT-cell involvement is strongest in the brain, liver, and hypoxic kidney, while its direct mechanistic role in cardiac IRI remains insufficiently defined. Thus, functional outcome is determined not by cell identity alone, but by the organ microenvironment, disease phase, activation pathway, and cellular subset.

## 5 Therapeutic potential of unconventional T cells

**Figure 6:**
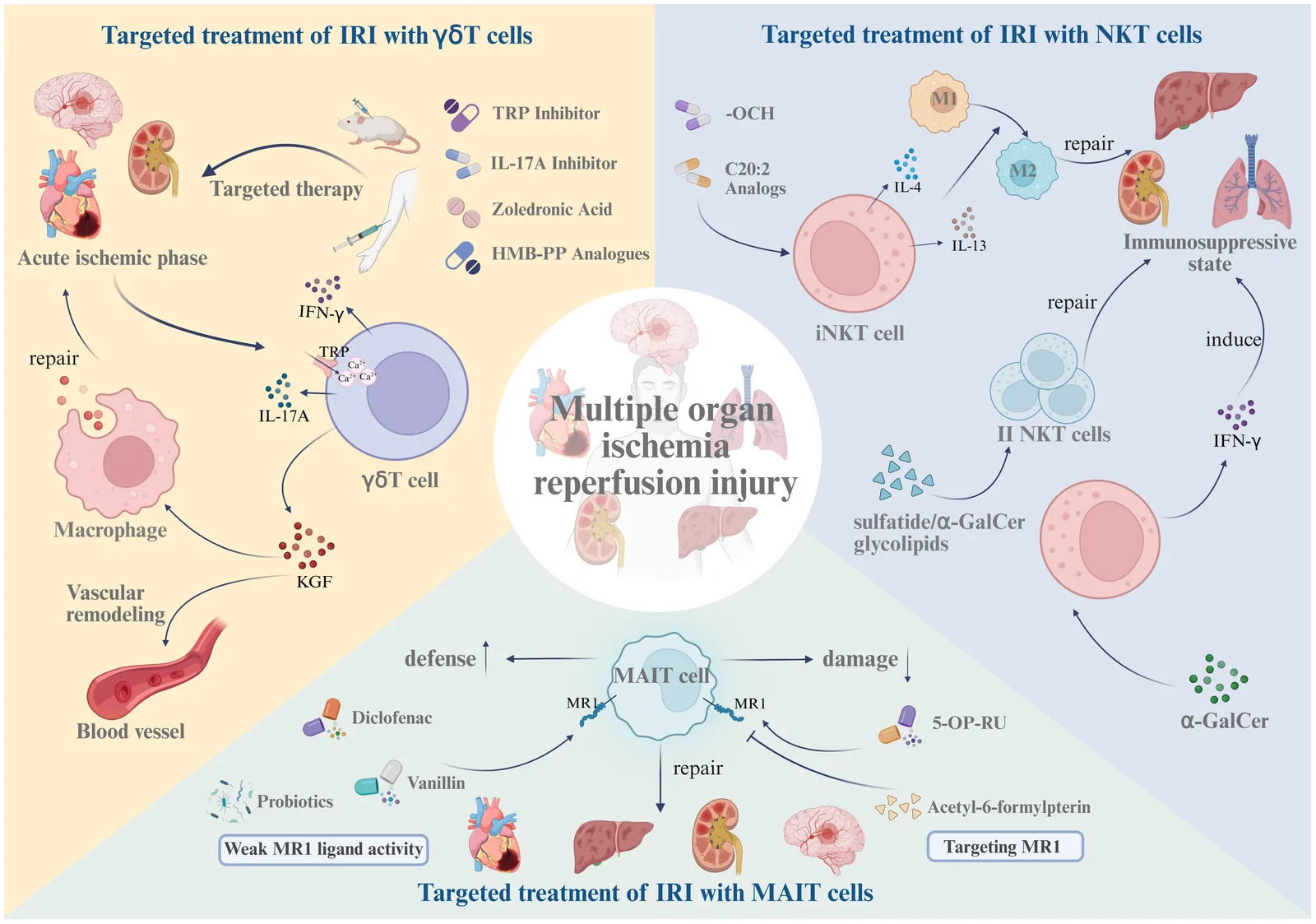
Intervention strategies targeting unconventional T cells in IRI. During the acute phase of rodent IRI, γδ T cells are activated through DAMP- and cytokine-driven pathways and produce IL-17A and/or IFN-γ. In this process, HMGB1-TLR4-induced IL-23 signaling further amplifies the IL-17A response, facilitating neutrophil infiltration, inflammatory expansion, oxidative stress, and apoptosis, aggravating tissue injury in multiple organs. In some contexts, enhanced TRP channel-associated Ca^2+^ influx in γδ T cells, promotes IL-17A and/or IFN-γ production, neutrophil recruitment. Accordingly, anti-γδ TCR antibodies, anti-IL-17A antibodies, and IL-23 neutralization strategies can mitigate secondary injury. During the repair phase, γδ T cells secrete KGF, VEGF, and IL-22, activating the PI3K-AKT and STAT3 proliferative pathways in target tissue cells, enhancing endothelial cell proliferation, VEGFR2 phosphorylation, capillary remodeling, and clearance of necrotic debris, and inhibiting M1 macrophage accumulation. Simultaneously, NKT cells can be regulated through α-GalCer analogs, IL-30, sulfatide, IL-33/ST2 blockade, or Tim-3 regulation to stimulate IL-4, IL-13, or IL-10 expression and enhance M2 polarization. MAIT cells can be regulated through MR1 agonists or antagonists and through the regulation of the gut microbiota.

Unconventional T cells provide several potential therapeutic entry points for IRI. However, their clinical utilization remains limited by various factors, including the precision of target molecules, the feasibility of translational pathways, and the need for systematic evaluation of patient-specific immune phenotypes. Rather than viewing γδ T cells, NKT cells, and MAIT cells as uniformly targetable populations, current evidence supports a more selective strategy. Among the proposed interventions, acute-phase suppression of pathogenic γδ T cell/IL-17A-related inflammatory signaling currently appears to have the strongest preclinical support. By contrast, NKT-cell-directed ligand modulation represents a mechanistically attractive but more context-dependent approach, whereas MAIT-cell-targeted therapy remains exploratory and requires further validation in clinically relevant IRI models (**Figure 6**).

The most promising strategy is the short-term inhibition of γδ T cell-driven inflammatory amplification during the acute reperfusion phase. In experimental stroke, IL-17A-producing γδ T cells, including Vγ4^+^ γδ T cells where specifically reported, amplify inflammatory cascades by promoting astrocytic CXCL1 expression and neutrophil recruitment, thereby exacerbating secondary brain injury (47, 49). In myocardial IRI, IL-17A contributes to cardiomyocyte apoptosis and neutrophil infiltration (147). Consequently, blocking the pathogenic activity of γδ T cells constitutes a therapeutic strategy for addressing the acute phase of IRI. TRP-channel-mediated Ca²^+^ signaling may contribute to the pathogenic activation of γδ T cells during the acute phase of IRI (148, 149). TRP channel blockers may increase the activation threshold of γδ T cells and diminish Ca^2+^ entry, consequently attenuating IL-17A-driven neutrophil recruitment and secondary injury during IRI. However, direct evidence linking TRP-channel blockade to reduced γδ T cell pathogenicity in IRI remains limited and warrants further investigation. In a mouse model of hepatic IRI, administration of anti-IL-17A antibodies or anti-γδTCR antibodies markedly reduced neutrophil infiltration and inflammatory amplification, and lowered tissue oxidative damage and cell apoptosis (88). In a mouse model of renal IRI, HMGB1-TLR4-induced IL-23 signaling further activated γδ T cells to produce IL-17A; neutralizing IL-23 or IL-17A similarly ameliorated renal dysfunction and mitigated injury (107). Therefore, among unconventional T-cell-based interventions, the γδ T cell/IL-17A axis should be prioritized for further translational development, particularly for early reperfusion-associated sterile inflammation.However, this strategy also has clear limitations. γδ T cells are not exclusively pathogenic. In the recovery phase, γδ T cells facilitate endothelial cell proliferation by secreting growth factors and modulating macrophage polarization to inhibit M1-type inflammatory responses (123). The depletion of γδ T cells results in decreased VEGFR2 phosphorylation in endothelial cells, reduced endothelial proliferation, decreased capillary density, and the concomitant accumulation of M1-type macrophages (123). Therefore, considering the dual role of γδ T cells in IRI, single-target interventions frequently have limitations. A more promising strategy involves bidirectional modulation: short-term inhibition of the IL-17A peak during the acute phase, followed by specific activation of γδ T cells in the repair stage using aminobisphosphonates (zoledronic acid) or HMB-PP analogs. This activation stimulates the release of substantial amounts of KGF, VEGF, and IL-22, activating PI3K-AKT and STAT3 proliferation pathways in tissue target cells, enhancing antimicrobial defense and clearance of necrotic debris, and accelerating tissue regeneration (150, 151). Furthermore, previous experiments have demonstrated that mesenchymal stem cells preconditioned with IL-17A exhibit significantly enhanced reparative capacity, indicating that moderate suppression of IL-17A secretion by γδ T cells may actually benefit tissue repair (152, 153).

The therapeutic potential of NKT cells in IRI has moderate preclinical support but greater biological complexity. α-GalCer activation can stimulate iNKT cells to produce IFN-γ, thereby maintaining an immunosuppressive condition in hepatic and renal IRI (154). Altering the glycolipid ligand—such as employing sphingosine chain-shortened analogs (OCH, C20:2) or co-administering cytokines such as IL-30—redirects iNKT cells toward a Th2-type cytokine profile (IL-4 and IL-13) (155). This subsequently promotes macrophage polarization toward the M2 reparative phenotype (81). In contrast, in mouse models of hepatic and pulmonary IRI, sulfatide-specific activation of type II NKT cells suppresses IFN-γ release from iNKT cells and induces IL-10 production and drives macrophage polarization toward the M2 phenotype, significantly reducing tissue damage (75, 154). Therefore, therapeutic strategies should not aim to indiscriminately inhibit the entire NKT-cell compartment. A more rational approach would be to suppress injurious iNKT-driven inflammatory amplification while preserving type II NKT-mediated immunoregulation, macrophage reprogramming, and tissue repair. Additionally, blocking the IL-33/ST2 signaling axis or targeting the Tim-3 immune checkpoint can inhibit NKT activation, providing new combination strategies to control sterile inflammation in the acute phase of IRI (156, 157). Approaches such as sulfatide-mediated activation of type II NKT cells, modified glycolipid ligands that skew cytokine production toward a Th2/regulatory profile, or blockade of upstream inflammatory signals such as the IL-33/ST2 axis may be useful, but these strategies remain largely preclinical and require careful assessment of dose, timing, organ context, and off-target immune effects.

Research on MAIT cells in IRI therapy is still at an early stage, which should currently be considered exploratory (70, 158). Although MAIT cells are increasingly recognized as responsive to sterile inflammatory signals and hypoxic tissue stress, the evidence for direct therapeutic targeting in IRI remains limited. Current intervention strategies targeting MAIT cells mostly focus on agonizing or antagonizing MR1 ligands. For example, microbial-derived vitamin B2 metabolites, including 5-OP-RU, can specifically bind to MR1 residues, thereby activating MAIT cells and enhancing host defense under immunosuppressive conditions (159). Conversely, small-molecule MR1 antagonists such as acetyl-6-formylpterin could theoretically block excessive MAIT activation to mitigate acute injury (160). Several commonly used drugs have demonstrated potential interactions with the MR1-MAIT axis. For instance, certain clinically available drugs—such as the non-steroidal anti-inflammatory drug diclofenac and its metabolites and vanillin- possess weak MR1 ligand activity, hinting at the possibility of repurposing old drugs (133, 161). Furthermore, modifying the gut microbial community with antibiotics or specific probiotics (Lactobacillus or Bifidobacterium) might reduce intestinal levels of microbial-derived vitamin B2 metabolites such as 5-OP-RU, leading to decreased MAIT cell numbers or suppressed function (162). Nevertheless, several barriers limit immediate translation. First, MAIT-cell biology differs between mice and humans, and MAIT cells are markedly less abundant in mice than in humans, making preclinical findings difficult to extrapolate (163). Second, MAIT cells may exert different functions depending on the organ, microbial exposure, cytokine milieu, and timing after reperfusion. Third, currently available MR1 ligands and drug-like modulators require more rigorous validation for specificity, pharmacokinetics, and safety in IRI settings. Therefore, MAIT-cell interventions should be framed as hypothesis-generating rather than ready for near-term clinical application.

## 6 Summary and outlook

IRI represents a complex immunopathological process in which immune activation, metabolic disturbance, and tissue repair are dynamically interconnected. This review reevaluates the significant immune functions of γδ T cells, NKT cells, and MAIT cells during the acute phase of reperfusion. Rather than functioning as uniformly pathogenic or protective populations, these cells exhibit highly context-dependent responses shaped by tissue microenvironment, disease stage, antigen availability, and metabolic signals. Understanding this functional plasticity provides new perspectives for deciphering sterile inflammation and immune regulation after ischemic injury.

However, several important limitations currently hold back the clinical translation of unconventional T cell–based strategies. First, most of our mechanistic understanding of these cells in IRI comes from experimental animal models, yet the abundance, distribution, TCR repertoire, and activation mechanisms of γδ T cells, NKT cells, and MAIT cells can differ substantially between rodents and humans. It remains unclear, therefore, how safely findings from preclinical models can be applied to human IRI. Second, although a growing body of clinical evidence now links changes in unconventional T cells to disease outcomes, human studies are still relatively sparse—especially those that trace how individual subsets shift over time through different phases of IRI. Third, therapeutic targeting of unconventional T cells remains challenging because of their functional heterogeneity. Efforts to suppress pathogenic subsets can all too easily compromise the protective functions that contribute to tissue repair and regeneration. The development of subset-specific interventions requires improved identification of molecular markers, spatial distribution patterns, and activation states within distinct tissue contexts.

Based on this comprehensive analysis of the molecular regulatory mechanisms governing their “double-edged sword” effects, future clinical applications should extend beyond merely developing therapeutic targets and should also advance early diagnostic and stratification strategies predicated on specific unconventional T cell responses. Rather than pursuing broad immune suppression, future therapeutic approaches should aim to achieve temporally controlled and subset-selective immune modulation. This strategy—integrating dynamic characteristics, molecular mechanism analysis, and precision immune monitoring—may provide a foundation for future translational investigations bridging mechanistic discoveries and clinical applications. Additionally, it provides a more targeted basis for decision-making in the development of personalized IRI intervention strategies. Although unconventional T cells represent promising candidates for precision immunotherapy and biomarker development, their clinical application remains at an exploratory stage and requires further validation before translation into effective human therapies.
